# Real-world evaluation of care for type 2 diabetes in Malaysia: A cross-sectional analysis of the treatment adherence to guideline evaluation in type 2 diabetes (TARGET-T2D) study

**DOI:** 10.1371/journal.pone.0296298

**Published:** 2024-01-02

**Authors:** Lee-Ling Lim, Zanariah Hussein, Nurain Md Noor, Anis S. Abd Raof, Norlaila Mustafa, Mohamed B. Long Bidin, Rohana Abdul Ghani, Syahrizan Samsuddin, Sy-Liang Yong, Siew-Hui Foo, Kavitha Raghuram, Payiarat Suwannasri, Wan Mohamad W. B., Thiam-Kian Chiew, Siew-Pheng Chan

**Affiliations:** 1 Faculty of Medicine, Department of Medicine, University of Malaya, Kuala Lumpur, Malaysia; 2 Department of Medicine and Therapeutics, The Chinese University of Hong Kong, Prince of Wales Hospital, Hong Kong, SAR; 3 Asia Diabetes Foundation, Shatin, Hong Kong, SAR; 4 Department of Medicine, Hospital Putrajaya, Putrajaya, Malaysia; 5 Faculty of Medicine, Department of Medicine, Universiti Kebangsaan Malaysia, Selangor, Malaysia; 6 Department of Medicine, Hospital Kuala Lumpur, Kuala Lumpur, Malaysia; 7 Faculty of Medicine, Department of Medicine, Universiti Teknologi MARA, Selangor, Malaysia; 8 Department of Medicine, Hospital Serdang, Selangor, Malaysia; 9 Department of Medicine, Hospital Tengku Ampuan Rahimah, Selangor, Malaysia; 10 Department of Medicine, Hospital Selayang, Selangor, Malaysia; 11 Boehringer Ingelheim Singapore Pte. Ltd, Singapore; 12 School of Medical Sciences, Universiti Sains Malaysia, Kelantan, Malaysia; 13 Faculty of Computer Science and Information Technology, Department of Software Engineering, University of Malaya, Kuala Lumpur, Malaysia; HT Ong Heart Clinic, MALAYSIA

## Abstract

**Aim:**

Given a lack of data on diabetes care performance in Malaysia, we conducted a cross-sectional study to understand the clinical characteristics, control of cardiometabolic risk factors, and patterns of use of guideline-directed medical therapy (GDMT) among patients with type 2 diabetes (T2D), who were managed at publicly-funded hospitals between December 2021 and June 2022.

**Methods:**

Patients aged ≥18 years with T2D from eight publicly-funded hospitals in the Greater Kuala Lumpur region, who had ≥2 outpatient visits within the preceding year and irrespective of treatment regimen, were eligible. The primary outcome was ≥2 treatment target attainment (defined as either HbA_1c_ <7.0%, blood pressure [BP] <130/80 mmHg, or low-density lipoprotein cholesterol [LDL-C] <1.8 mmol/L). The secondary outcomes were the individual treatment target, a combination of all three treatment targets, and patterns of GDMT use. To assess for potential heterogeneity of study findings, all outcomes were stratified according to prespecified baseline characteristics namely 1) history of atherosclerotic cardiovascular disease (ASCVD; yes/no) and 2) clinic type (Diabetes specialist versus General medicine).

**Results:**

Among 5094 patients (mean±SD age 59.0±13.2 years; T2D duration 14.8±9.2 years; HbA_1c_ 8.2±1.9% (66±21 mmol/mol); BMI 29.6±6.2 kg/m^2^; 45.6% men), 99% were at high/very high cardiorenal risk. Attainment of ≥2 treatment targets was at 18%, being higher in General medicine than in Diabetes specialist clinics (20.8% versus 17.5%; p = 0.039). The overall statin coverage was 90%. More patients with prior ASCVD attained LDL-C <1.4 mmol/L than those without (13.5% versus 8.4%; p<0.001). Use of sodium-glucose cotransporter-2 (SGLT2) inhibitors (13.2% versus 43.2%), glucagon-like peptide-1 receptor agonists (GLP1-RAs) (1.0% versus 6.2%), and insulin (27.7% versus 58.1%) were lower in General medicine than in Diabetes specialist clinics.

**Conclusions:**

Among high-risk patients with T2D, treatment target attainment and use of GDMT were suboptimal.

## 1. Introduction

Type 2 diabetes (T2D) is one of the major public health concerns in Malaysia. Based on the Malaysian National Health and Morbidity Survey in 2019, the prevalence of diabetes in adults aged ≥18 years has increased from 11.2% in 2011 to 18.3% [1]. This brings the total number of adults living with diabetes to 3.9 million [1]. Key cardiometabolic risk factors namely T2D, hypertension, and dyslipidaemia often occur together. To date, 3.4 million patients have two or more of these risk factors [1]. This highlights the importance of multicomponent interventions in managing patients with T2D to mitigate the long-term risk of complications and improve quality of life [2].

The increasing burden of T2D and its complications imposes substantial healthcare costs (both direct and indirect), especially when the healthcare system is heavily subsidized by the Malaysian government [3,4]. Although atherosclerotic cardiovascular disease (ASCVD) and heart failure are leading causes of disability and premature death, the 2019 GBD-NHLBI-JACC Global Burden of Cardiovascular Diseases Study reported the lack of improvement in mitigating these risks on a global level in the past three decades [5]. High systolic BP, high low-density lipoprotein cholesterol (LDL-C), high body mass index (BMI), high fasting plasma glucose, and kidney dysfunction remain the top five modifiable risk factors for ASCVD and heart failure between 1990 and 2019 [5].

Large-scale randomized clinical trials reported that sustained reduction of cardiometabolic risk factors for 2–5 years improved clinical outcomes in patients with T2D [2]. Several meta-analyses had also reported that reduction of HbA_1c_ by 0.9% (10 mmol/mol) [6,7], systolic BP by 10 mmHg [8], and LDL-C by 1 mmol/L [9] independently reduced the risk of ASCVD and/or all-cause death by 10–20%. Since the landmark EMPA-REG OUTCOME trial in 2015 [10], several randomized clinical trials with sodium-glucose cotransporter-2 (SGLT2) inhibitors confirmed the marked reduction in risks for ASCVD, heart failure, and kidney dysfunction in patients with T2D and even improved cardiorenal outcomes in patients without T2D [11–17]. Glucagon-like peptide-1 receptor agonists (GLP1-RAs) have also shown risk reductions in ASCVD and kidney dysfunction in patients with T2D [18–22]. These findings have led to a changing landscape of the management of T2D [23,24].

Given its high burden and the paradigm shift in T2D management, evidence-based consensus guidance and new quality indicators have been developed for increasing the efficiency of care delivery. In Malaysia, the National Diabetes Registry was established in 2009 to evaluate the variations in care delivery and to uncover opportunities for quality improvement at publicly-funded primary care clinics [25]. However, similar mechanisms in hospital-based settings have thus far been limited.

## 2. Methods

### 2.1. Study design and population

**Fig 1:**
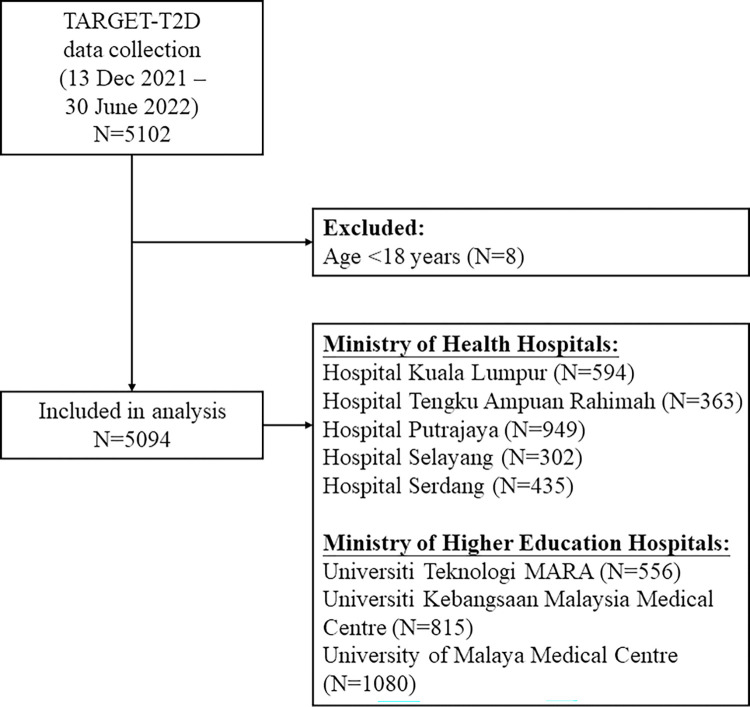
Study flow diagram.

The TARGET-T2D study is the first, large-scale quality improvement initiative for understanding the care patterns of patients with T2D in hospital-based settings in Malaysia. This was a cross-sectional study to describe the clinical characteristics, control of cardiometabolic risk factors, and patterns of medication use at eight publicly-funded specialist hospitals in the Greater Kuala Lumpur region, the capital of Malaysia. Three hospitals were academic institutions under the Ministry of Higher Education (MOHE) whilst the remaining hospitals were managed by the Ministry of Health (MOH) (Fig 1).

We included patients aged ≥18 years with T2D treated with oral glucose-lowering drugs and/or injectable therapy, who had at least two outpatient visits at either Diabetes specialist or General medicine clinics within the preceding year of data collection. We excluded patients with 1) type 1 diabetes, defined as a presentation with either diabetic ketoacidosis, unprovoked ketosis, or continuous insulin requirement within 12 months of diagnosis; 2) gestational diabetes mellitus, and 3) secondary diabetes mellitus.

We used the convenience sampling method and conducted data collection at all study sites from 13 December 2021 to 30 June 2022 for a total duration of six months. Before each clinic day, the study team prepared the outpatient appointment lists to facilitate data collection. The study team was trained to extract relevant data (including sociodemographic, comorbidities, medications, anthropometric, and laboratory measurements) from health records (either electronic or manual, depending on the site facility). We standardized data collection using established definitions, uniform data entry, and periodic data quality assurance by the Steering Committee of the study. We developed a TARGET-T2D web portal in collaboration with the Department of Software Engineering, University of Malaya. All data were pseudonymized and stored in the web portal in a manner compliant with local regulations. Any data not meeting predefined clinical plausibility thresholds were flagged for manual review with each study site.

The present study was approved by the Medical Research & Ethics Committee, Ministry of Health (NMRR ID-21-02100-BPE [IIR]) and three MOHE institutional ethics review boards. Given that there was no data collection beyond that of routine care, a waiver of written informed consent was granted.

### 2.2. Study outcomes

The primary outcome was the proportion of patients with T2D attaining ≥2 treatment targets, defined as 1) HbA_1c_ <7.0% (53 mmol/mol); 2) BP <130/80 mmHg; and 3) LDL-C <1.8 mmol/L [23,26–28]. Secondary outcomes were the individual target, a combination of all three targets, and medication patterns. To assess for potential heterogeneity of results, all study outcomes were stratified according to prespecified baseline characteristics namely prior ASCVD and clinic type (Diabetes specialist versus General medicine).

Based on the recommendations of the Malaysian Clinical Practice Guideline of Type 2 Diabetes [23], we recorded glycaemic parameters namely fasting plasma glucose and HbA_1c_ that were available up to six months prior to data collection. For non-glycaemic parameters namely lipid profile (including total cholesterol, LDL-C, triglyceride, and high-density lipoprotein cholesterol [HDL-C]), kidney function, liver function, and albuminuria, we recorded these measurements up to 12 months prior to data collection. To standardize the definition of albuminuria, we converted the values of urinary protein:creatinine ratio to urinary albumin:creatinine ratio (ACR) based on the equation developed by the CKD Prognosis Consortium [29]. We used the most recent laboratory measurements to define study outcomes.

### 2.3. Statistical analysis

Data were presented as mean±standard deviation (SD) or median (interquartile range [IQR]) for continuous variables with either normal or skewed distribution, respectively. We assessed for normality of data using the histograms, QQ plots, Shapiro Wilk, or Kolmogorov Smirnov test. We logarithmically transformed continuous variables with skewed distributions for analysis. Categorical variables were presented as numbers and percentages. Patients with T2D were categorized into either moderate, high, or very high cardiovascular risk based on the 2019 European Society of Cardiology (ESC) risk criteria [26].

For the two-group comparison of continuous variables, we used an independent *t*-test for data with normal distributions whilst Wilcoxon rank-sum test was for data with skewed distributions. We used the Chi-square test for between-group comparisons of categorical variables. We performed subgroup analyses, stratified by history of ASCVD and clinic type (Diabetes specialist versus General medicine).

We performed pairwise deletion for variables with missing values. All analyses were performed using R 4.2.1 [30]. A 2-tailed p-value <0.05 denoted statistical significance.

## 3. Results

Fig 1 describes the study flow. Among 5102 patients with T2D, we included 5094 patients aged ≥18 years in the present analysis, of whom 45.6% were men. Table 1 shows the baseline characteristics of the overall cohort, stratified by ASCVD status. The cohort was predominantly of Malay ethnicity (58.2%), followed by Indian (23.6%) and Chinese (18.2%). Fewer than 10% of them were current smokers.

**Table 1:** Baseline characteristics of patients with type 2 diabetes in the TARGET-T2D study, stratified by atherosclerotic cardiovascular disease (ASCVD) status.

	Entire cohort (n = 5094)		Without ASCVD (n = 3523)		With ASCVD (n = 1495)		p-value
Age at hospital visit, year	5094	59.0±13.2	3523	57.3±13.9	1495	63.1±10.4	<0.001
Duration of diabetes, year	5087	14.8±9.2	3519	13.9±8.8	1494	16.8±9.6	<0.001
Men, n (%)	5094	2323 (45.6%)	3523	1382 (39.2%)	1495	910 (60.9%)	<0.001
Ethnicity, n (%)	5047		3490		1481		<0.001
Chinese		921 (18.2%)		660 (18.9%)		258 (17.4%)	
Indian		1189 (23.6%)		722 (20.7%)		459 (31.0%)	
Malay		2937 (58.2%)		2108 (60.4%)		764 (51.6%)	
Clinic type, n (%)	5093		3523		1494		<0.001
Diabetes specialist		4170 (81.9%)		2947 (83.7%)		1147 (76.8%)	
General medicine		923 (18.1%)		576 (16.3%)		347 (23.2%)	
Education level, n (%)	4790		3345		1376		<0.001
No formal		168 (3.5%)		105 (3.1%)		62 (4.5%)	
Others		11 (0.2%)		9 (0.3%)		2 (0.1%)	
Primary		483 (10.1%)		302 (9.0%)		179 (13.0%)	
Secondary		2110 (44.1%)		1454 (43.5%)		636 (46.2%)	
Tertiary		2018 (42.1%)		1475 (44.1%)		497 (36.1%)	
Family history of diabetes, n (%)	4830	3648 (75.5%)	3336	2543 (76.2%)	1421	1047 (73.7%)	0.067
Current smoker, n (%)	4820	352 (7.3%)	3324	214 (6.4%)	1425	133 (9.3%)	0.001
Regular alcohol drinker, n (%)	3554	38 (1.1%)	2412	22 (0.9%)	1071	16 (1.5%)	0.177
**Cardiometabolic risk factors**							
Fasting plasma glucose, mmol/L	4577	8.2±3.6	3190	8.2±3.6	1321	8.3±3.6	0.490
HbA_1c_ (NGSP, %)	4640	8.2±1.9	3222	8.1±1.9	1344	8.3±1.9	0.027
HbA_1c_ (IFCC, mmol/mol)	4640	66±21	3222	65±21	1344	67±21	0.027
Systolic BP, mmHg	5090	139.4±18.3	3522	139±18.1	1494	140.4±18.9	0.018
Diastolic BP, mmHg	5053	77.0±11.3	3499	77.5±11.2	1480	75.7±11.6	<0.001
Total cholesterol, mmol/L	4837	4.5±1.3	3345	4.5±1.2	1418	4.3±1.3	<0.001
LDL-C, mmol/L	4767	2.5±1.0	3290	2.5±1.0	1403	2.3±1.0	<0.001
Non-HDL-C, mmol/L	4816	3.2±1.2	3331	3.3±1.2	1411	3.1±1.2	<0.001
HDL-C, mmol/L	4819	1.2±0.3	3335	1.3±0.4	1410	1.2±0.3	<0.001
Triglyceride^#^, mmol/L	4836	1.5 (1.1–2.0)	3347	1.5 (1.1–2.0)	1415	1.5 (1.1–2.0)	0.671
BMI, kg/m^2^	4932	29.6±6.2	3440	29.8±6.4	1424	28.9±5.6	<0.001
Waist (male), cm	2134	99.7±13.8	1276	100±14.1	833	99.1±13.2	0.140
Waist (female), cm	2525	95.1±12.7	1975	95.2±12.6	513	94.6±13.3	0.328
eGFR, mL/min/1.73m^2^	4873	75.3±29.3	3360	79.1±29.1	1440	66.4±27.8	<0.001
Urinary ACR^#^, mg/mmol	3112	3.3 (1.1–11.0)	2167	3 (1.1–9.0)	894	4.5 (1.2–22.2)	<0.001
**Comorbidities, n (%)**							
BMI <25 kg/m^2^	4932	1129 (22.9%)	3440	774 (22.5%)	1424	340 (23.9%)	0.316
Waist ≥90 cm (male)	2134	1649 (77.3%)	1276	981 (76.9%)	833	647 (77.7%)	0.712
Waist ≥80 cm (female)	2525	2322 (92.0%)	1975	1820 (92.2%)	513	466 (90.8%)	0.379
ASCVD	5018	1495 (29.8%)					
HHF	4994	191 (3.8%)	3521	59 (1.7%)	1463	132 (9.0%)	<0.001
eGFR <60 mL/min/1.73m^2^	5062	1511 (29.8%)	3495	892 (25.5%)	1493	597 (40.0%)	<0.001
Urinary ACR >3 mg/mmol	3801	2228 (58.6%)	2669	1525 (57.1%)	1079	678 (62.8%)	0.002
ESC 2019 cardiovascular risk category	4782		3240		1495		<0.001
Moderate		8 (0.2%)		8 (0.2%)		0 (0%)	
High		70 (1.5%)		70 (2.2%)		0 (0%)	
Very high		4704 (98.4%)		3162 (97.6%)		1495 (100%)	
**Treatment targets, n (%)**							
HbA_1c_ <7% (53 mmol/mol)	4640	1378 (29.7%)	3222	986 (30.6%)	1344	367 (27.3%)	0.029
HbA_1c_ <8.5% (69 mmol/mol)	4640	3014 (65.0%)	3222	2116 (65.7%)	1344	846 (62.9%)	0.084
BP <130/80 mmHg	5066	1157 (22.8%)	3507	783 (22.3%)	1485	356 (24.0%)	0.219
LDL-C <1.4 mmol/L	4767	470 (9.9%)	3290	275 (8.4%)	1403	190 (13.5%)	<0.001
LDL-C <1.8 mmol/L	4767	1253 (26.3%)	3290	779 (23.7%)	1403	458 (32.6%)	<0.001
LDL-C <2.6 mmol/L	4767	3030 (63.6%)	3290	1998 (60.7%)	1403	989 (70.5%)	<0.001
≥2 treatment targets^¥^	4465	803 (18.0%)	3096	538 (17.4%)	1297	254 (19.6%)	0.091
All 3 treatment targets^¥^	4465	114 (2.6%)	3096	64 (2.1%)	1297	48 (3.7%)	0.002
≥2 treatment targets (audit)^γ^	4465	2385 (53.4%)	3096	1620 (52.3%)	1297	726 (56.0%)	0.029
All 3 treatment targets (audit)^γ^	4465	512 (11.5%)	3096	328 (10.6%)	1297	178 (13.7%)	0.004
**Medications**							
**Glucose-lowering, n (%)**							
Insulin	5094	2678 (52.6%)	3523	1808 (51.3%)	1495	831 (55.6%)	0.006
Metformin	5094	4311 (84.6%)	3523	3060 (86.9%)	1495	1183 (79.1%)	<0.001
Sulphonylurea	5094	1306 (25.6%)	3523	958 (27.2%)	1495	331 (22.1%)	<0.001
DPP4 inhibitors	5094	2139 (42.0%)	3523	1487 (42.2%)	1495	606 (40.5%)	0.285
SGLT2 inhibitors	5094	1924 (37.8%)	3523	1258 (35.7%)	1495	631 (42.2%)	<0.001
GLP1-RA	5094	268 (5.3%)	3523	209 (5.9%)	1495	55 (3.7%)	0.001
**BP-lowering**							
RAS inhibitors	5094	3349 (65.7%)	3523	2224 (63.1%)	1495	1080 (72.2%)	<0.001
ARNI	5094	22 (0.4%)	3523	1 (0%)	1495	21 (1.4%)	<0.001
Beta-blockers	5094	1650 (32.4%)	3523	730 (20.7%)	1495	903 (60.4%)	<0.001
CCB	5094	2296 (45.1%)	3523	1571 (44.6%)	1495	683 (45.7%)	0.496
MRA	5094	128 (2.5%)	3523	40 (1.1%)	1495	88 (5.9%)	<0.001
Diuretics	5094	933 (18.3%)	3523	559 (15.9%)	1495	365 (24.4%)	<0.001
Alpha-blockers	5094	198 (3.9%)	3523	111 (3.2%)	1495	86 (5.8%)	<0.001
**Lipid-lowering**							
Statins	5094	4558 (89.5%)	3523	3066 (87.0%)	1495	1422 (95.1%)	<0.001
Fenofibrate	5094	583 (11.4%)	3523	400 (11.4%)	1495	179 (12.0%)	0.562
Ezetimibe	5094	265 (5.2%)	3523	116 (3.3%)	1495	146 (9.8%)	<0.001
**Antiplatelet**	5094	1738 (34.1%)	3523	425 (12.1%)	1495	1299 (86.9%)	<0.001

Footnotes: Results are presented as mean±standard deviation, ^#^median (interquartile range), or number (percentage), as appropriate. ^¥^Treatment targets were based on HbA_1c_ <7% (53 mmol/mol), blood pressure (BP) <130/80 mmHg, and low-density lipoprotein cholesterol (LDL-C) <1.8 mmol/L. ^γ^Treatment targets were based on the audit criteria of the 2020 Malaysian Clinical Practice Guideline on Type 2 Diabetes, defined as HbA_1c_ <8.5% (69 mmol/mol), BP <130/80 mmHg, and LDL-C <1.8 mmol/L. ARNI, angiotensin receptor/neprilysin inhibitors; ASCVD, atherosclerotic cardiovascular disease; BMI, body mass index; CCB, calcium channel blockers; DPP4 inhibitors, dipeptidyl peptidase-4 inhibitors; ESC, European Society of Cardiology; GLP1-RAs, glucagon-like peptide-1 receptor agonists; HDL-C, high-density lipoprotein cholesterol; HHF, hospitalization for heart failure; MI, myocardial infarction; MRA, mineralocorticioid receptor antagonists; NGSP, National Glycohemoglobin Standardization Program; IFCC, International Federation of Clinical Chemistry; RAS inhibitors, renin-angiotensin system inhibitors; SGLT2 inhibitors, sodium-glucose cotransporter-2 inhibitors; Urinary ACR, urinary albumin:creatinine ratio.

**Fig 2:**
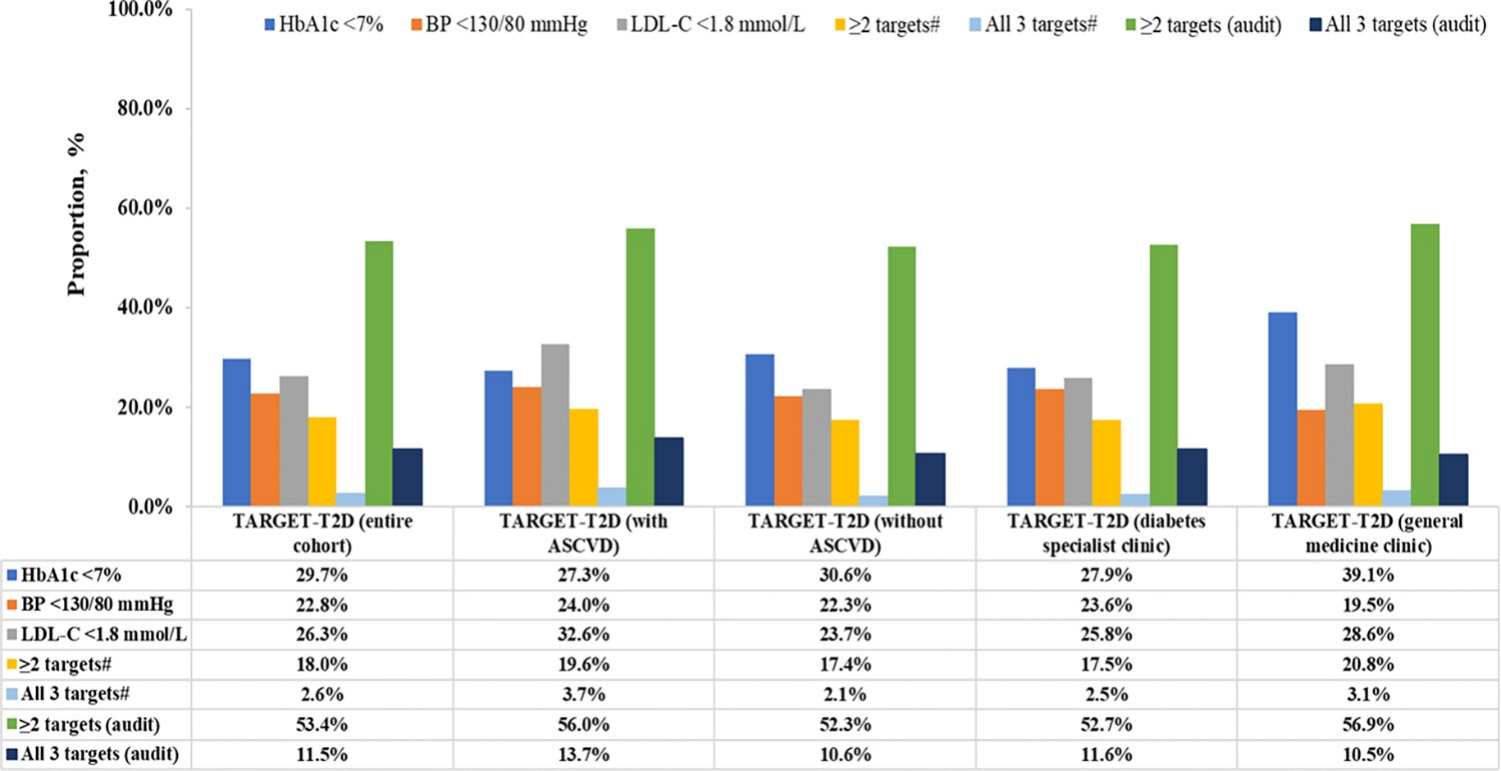
Treatment target attainment among patients with type 2 diabetes in the TARGET-T2D study, stratified by atherosclerotic cardiovascular disease (ASCVD) status and clinic type. ^#^Treatment targets were based on HbA_1c_ <7%, BP <130/80 mmHg, and LDL-C <1.8 mmol/L. “Audit” means treatment targets were based on the audit criteria of the 2020 Malaysian Clinical Practice Guideline on Type 2 Diabetes, defined as HbA_1c_ <8.5%, blood pressure (BP) <130/80 mmHg, and low-density lipoprotein cholesterol (LDL-C) <1.8 mmol/L.

In the entire cohort, the mean±SD HbA_1c_ was 8.2±1.9% (66±21 mmol/mol) and one-third attained HbA_1c_<7% (53 mmol/mol) at baseline. Compared with those without prior ASCVD, a lower proportion of patients with prior ASCVD attained HbA_1c_<7% (53 mmol/mol) (27.3% versus 30.6%; p = 0.029) (Table 1 and Fig 2). The proportion of patients who attained BP<130/80 mmHg was 22.8% with no significant difference by ASCVD status (Table 1 and Fig 2). Compared with those without prior ASCVD, a higher proportion of patients with prior ASCVD attained either LDL-C<1.4 mmol/L (13.5% versus 8.4%) or LDL-C<1.8 mmol/L (32.6% versus 23.7%) (p<0.001 for both; Table 1 and Fig 2). A total of 18% of the entire cohort attained ≥2 treatment targets, which was higher in the General medicine clinics compared with the Diabetes specialist clinics (20.8% versus 17.5%; p = 0.039) (Table 2 and Fig 2).

**Table 2:** Baseline characteristics of patients with type 2 diabetes in the TARGET-T2D study, stratified by clinic type.

	Diabetes specialist clinic (n = 4170)		General medicine clinic (n = 923)		p-value
Age at hospital visit, year	4170	58.7±13.4	923	60.5±12.0	<0.001
Duration of diabetes, year	4165	15.7±9.3	921	10.6±7.4	<0.001
Men, n (%)	4170	1852 (44.4%)	923	471 (51.0%)	<0.001
Ethnicity, n (%)	4136		910		<0.001
Chinese		733 (17.7%)		187 (20.5%)	
Indian		913 (22.1%)		276 (30.3%)	
Malay		2490 (60.2%)		447 (49.1%)	
Education level, n (%)	3926		864		<0.001
No formal		101 (2.6%)		67 (7.8%)	
Others		7 (0.2%)		4 (0.5%)	
Primary		369 (9.4%)		114 (13.2%)	
Secondary		1732 (44.1%)		378 (43.8%)	
Tertiary		1717 (43.7%)		301 (34.8%)	
Family history of diabetes, n (%)	4028	3038 (75.4%)	801	609 (76.0%)	0.749
Current smoker, n (%)	3920	276 (7.0%)	899	76 (8.5%)	0.162
Regular alcohol drinker, n (%)	3012	31 (1.0%)	541	7 (1.3%)	0.746
**Cardiometabolic risk factors**					
Fasting plasma glucose, mmol/L	3838	8.3±3.7	738	7.9±3.0	<0.001
HbA_1c_ (NGSP, %)	3902	8.2±1.9	737	7.9±1.9	<0.001
HbA_1c_ (IFCC, mmol/mol)	3902	66±21	737	63±21	<0.001
Systolic BP, mmHg	4166	139.1±18.6	923	140.6±17.1	0.023
Diastolic BP, mmHg	4138	76.6±11.2	914	78.7±11.5	<0.001
Total cholesterol, mmol/L	4005	4.5±1.3	831	4.4±1.2	0.050
LDL-C, mmol/L	3943	2.5±1.0	823	2.4±1.0	0.103
Non-HDL-C, mmol/L	3988	3.2±1.2	827	3.2±1.2	0.049
HDL-C, mmol/L	3992	1.2±0.3	826	1.2±0.4	0.759
Triglyceride^#^, mmol/L	4007	1.4 (1.1–2.0)	828	1.4 (1.1–1.9)	0.015
BMI, kg/m^2^	4022	29.7±6.3	909	29.2±5.8	0.045
Waist (male), cm	1712	100.6±13.9	422	96.1±12.7	<0.001
Waist (female), cm	2115	95.6±12.9	409	92.7±11.0	<0.001
eGFR, mL/min/1.73m^2^	4026	74.4±29.8	846	79.8±26.6	<0.001
Urinary ACR^#^, mg/mmol	2509	3 (0.9–13.6)	602	3 (1.9–7.0)	<0.001
**Comorbidities, n (%)**					
BMI <25 kg/m^2^	4022	927 (23.0%)	909	202 (22.2%)	0.623
Waist ≥90 cm (male)	1712	1360 (79.4%)	422	289 (68.5%)	<0.001
Waist ≥80 cm (female)	2115	1943 (91.9%)	409	379 (92.7%)	0.657
ASCVD	4094	1147 (28.0%)	923	347 (37.6%)	<0.001
HHF	4071	157 (3.9%)	922	34 (3.7%)	0.884
eGFR <60 mL/min/1.73m^2^	4026	1263 (31.4%)	846	209 (24.7%)	<0.001
Urinary ACR >3 mg/mmol	2509	1446 (57.6%)	602	399 (66.3%)	<0.001
ESC cardiovascular risk category	3898		883		0.019
Moderate		8 (0.2%)		0 (0%)	
High		65 (1.7%)		5 (0.6%)	
Very high		3825 (98.1%)		878 (99.4%)	
**Treatment targets, n (%)**					
HbA_1c_ <7% (53 mmol/mol)	3902	1090 (27.9%)	737	288 (39.1%)	<0.001
HbA_1c_ <8.5% (69 mmol/mol)	3902	2492 (63.9%)	737	521 (70.7%)	<0.001
BP <130/80 mmHg	4145	978 (23.6%)	920	179 (19.5%)	0.008
LDL-C <1.4 mmol/L	3943	392 (9.9%)	823	77 (9.4%)	0.654
LDL-C <1.8 mmol/L	3943	1017 (25.8%)	823	235 (28.6%)	0.111
LDL-C <2.6 mmol/L	3943	2474 (62.7%)	823	555 (67.4%)	0.012
≥2 treatment targets^¥^	3752	655 (17.5%)	712	148 (20.8%)	0.039
All 3 treatment targets^¥^	3752	92 (2.5%)	712	22 (3.1%)	0.390
≥2 treatment targets (audit)^γ^	3752	1979 (52.7%)	712	405 (56.9%)	0.047
All 3 treatment targets (audit)^γ^	3752	437 (11.6%)	712	75 (10.5%)	0.429
**Medications**					
**Glucose-lowering, n (%)**					
Insulin	4170	2422 (58.1%)	923	256 (27.7%)	<0.001
Metformin	4170	3515 (84.3%)	923	795 (86.1%)	0.176
Sulphonylurea	4170	943 (22.6%)	923	362 (39.2%)	<0.001
DPP4 inhibitors	4170	1996 (47.9%)	923	142 (15.4%)	<0.001
SGLT2 inhibitors	4170	1802 (43.2%)	923	122 (13.2%)	<0.001
GLP1-RAs	4170	259 (6.2%)	923	9 (1.0%)	<0.001
**BP-lowering**					
RAS inhibitors	4170	2741 (65.7%)	923	607 (65.8%)	1.000
ARNI	4170	20 (0.5%)	923	2 (0.2%)	0.409
Beta-blockers	4170	1332 (31.9%)	923	318 (34.5%)	0.151
CCB	4170	1857 (44.5%)	923	439 (47.6%)	0.102
MRA	4170	101 (2.4%)	923	27 (2.9%)	0.443
Diuretics	4170	797 (19.1%)	923	136 (14.7%)	0.002
Alpha-blockers	4170	149 (3.6%)	923	49 (5.3%)	0.018
**Lipid-lowering**					
Statins	4170	3720 (89.2%)	923	837 (90.7%)	0.207
Fenofibrate	4170	532 (12.8%)	923	51 (5.5%)	<0.001
Ezetimibe	4170	245 (5.9%)	923	20 (2.2%)	<0.001
**Antiplatelet**	4170	1371 (32.9%)	923	366 (39.7%)	<0.001

Footnotes: Results are presented as mean±standard deviation, ^#^median (interquartile range), or number (percentage), as appropriate. ^¥^Treatment targets were based on HbA_1c_ <7% (53 mmol/mol), blood pressure (BP) <130/80 mmHg, and low-density lipoprotein cholesterol (LDL-C) <1.8 mmol/L. ^γ^Treatment targets were based on the audit criteria of the 2020 Malaysian Clinical Practice Guideline on Type 2 Diabetes, defined as HbA_1c_ <8.5% (69 mmol/mol), BP <130/80 mmHg, and LDL-C <1.8 mmol/L. ARNI, angiotensin receptor/neprilysin inhibitors; ASCVD, atherosclerotic cardiovascular disease; BMI, body mass index; CCB, calcium channel blockers; DPP4 inhibitors, dipeptidyl peptidase-4 inhibitors; ESC, European Society of Cardiology; GLP1-RAs, glucagon-like peptide-1 receptor agonists; HDL-C, high-density lipoprotein cholesterol; HHF, hospitalization for heart failure; MRA, mineralocorticioid receptor antagonists; NGSP, National Glycohemoglobin Standardization Program; IFCC, International Federation of Clinical Chemistry; RAS inhibitors, renin-angiotensin system inhibitors; SGLT2 inhibitors, sodium-glucose cotransporter-2 inhibitors; Urinary ACR, urinary albumin:creatinine ratio.

There were high rates of general and central obesity (Table 1). The mean±SD BMI of the entire cohort was 29.6±6.2 kg/m^2^, wherein only 22.9% patients had a BMI of <25 kg/m^2^. The mean±SD waist circumference was 99.7±13.8 cm in males and 95.1±12.7 cm in females. One-third of the entire cohort had an eGFR <60 mL/min/1.73m^2^, whilst about 4% had prior hospitalization for heart failure. Compared with those without prior ASCVD, patients with prior ASCVD reported higher proportions of eGFR <60 mL/min/1.73m^2^ (40.0% versus 25.5%) and heart failure (9.0% versus 1.7%) (p<0.001 for both; Table 1). Of note, 99% of the entire cohort were at high-/very high cardiorenal risk according to the ESC 2019 classification, irrespective of clinic type (Tables 1 and 2).

**Fig 3:**
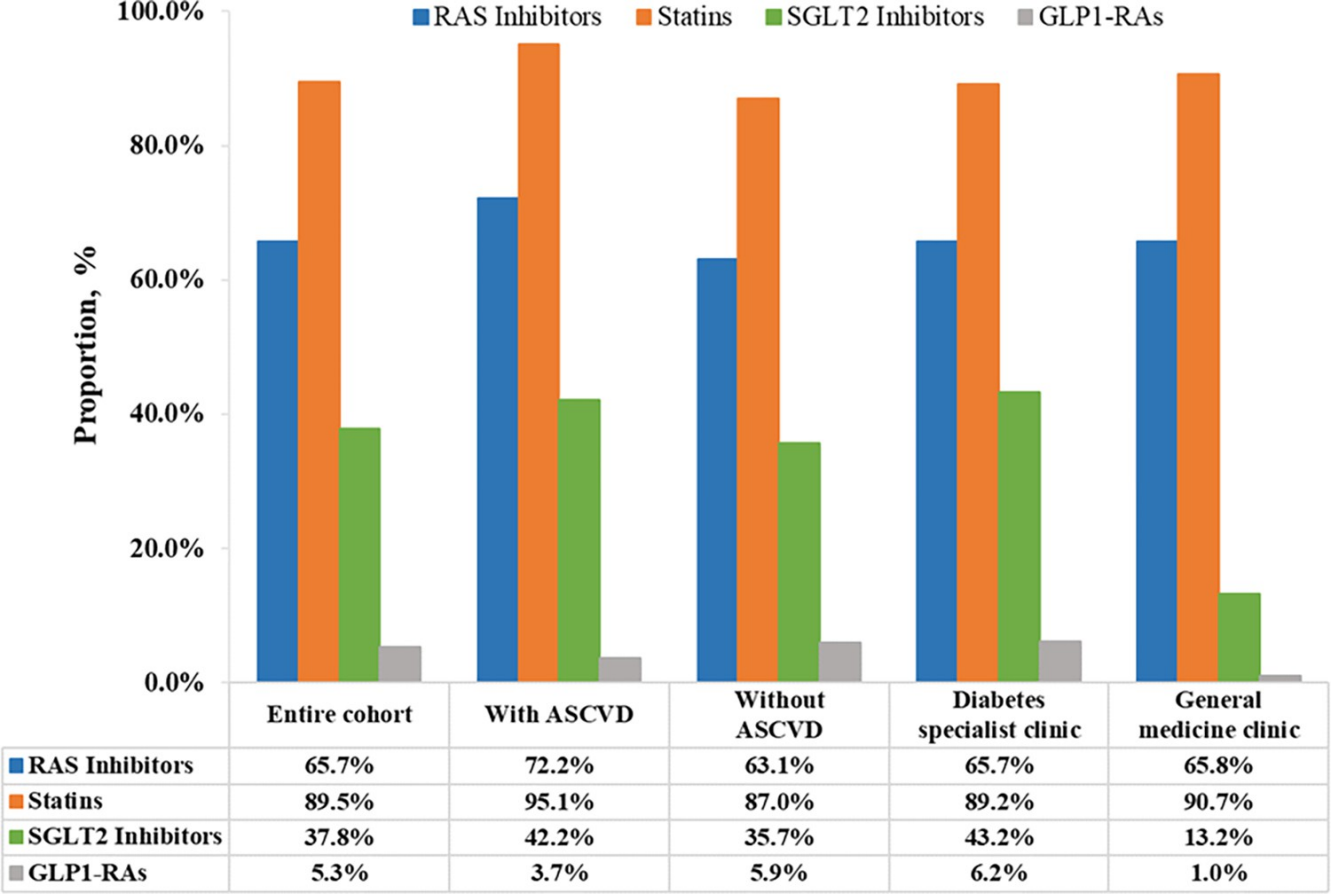
Use of guideline-directed medical therapies in the entire cohort of the TARGET-T2D study, stratified by atherosclerotic cardiovascular disease (ASCVD) status and clinic type. GLP1-RAs, glucagon-like peptide-1 receptor agonists; RAS inhibitors, renin-angiotensin system inhibitors; SGLT2 inhibitors, sodium-glucose cotransporter-2 inhibitors.

Compared with those without ASCVD, use of SGLT2 inhibitors (42.2% versus 35.7%; p<0.001) was significantly higher among patients with prior ASCVD, but not for GLP1-RAs (3.7% versus 5.9%) (Table 1 and Fig 3). Similar results were observed for RAS inhibitors (72.2% versus 63.1%), beta-blockers (60.4% versus 20.7%), statin therapy (95.1% versus 87.0%), and antiplatelet therapy (86.9% versus 12.1%) (p<0.001 for all; Table 1 and Fig 3). Of note, compared with the Diabetes specialist clinics, use of SGLT2 inhibitors (13.2% versus 43.2%), GLP1-RAs (1.0% versus 6.2%), DPP4 inhibitors (15.4% versus 47.9%), and insulin (27.7% versus 58.1%) were lower in the General medicine clinics (Table 2 and Fig 3). Regarding lipid-lowering medications, use of fenofibrate (5.5% versus 12.8%) and ezetimibe (2.2% versus 5.9%) were also lower at the General medicine clinics with no significant between-group differences in lipid profile. Use of BP-lowering medications was not significantly different between the Diabetes specialist and General medicine clinics.

Compared with those managed in the Diabetes specialist clinics, patients in the General medicine clinics were older (60.5±12.0 versus 58.7±13.4 years) with a shorter duration of T2D (10.6±7.4 versus 15.7±9.3 years) (Table 2). Both clinic types had similar cardiometabolic risk factors and comorbidity profiles, except for a lower HbA_1c_ level (7.9±1.9% (63±21 mmol/mol) versus 8.2±1.9% (66±21 mmol/mol)) and a higher proportion of ASCVD (37.6% versus 28.0%) and albuminuria (66.3% versus 57.6%) in the General medicine clinics. Compared with those managed in the Diabetes specialist clinics, a higher proportion of patients in the General medicine clinics attained HbA_1c_<7% (53 mmol/mol) (39.1% versus 27.9%) and ≥2 treatment targets (20.8% versus 17.5%).

## 4. Discussion

In this real-world cohort of >5000 patients with T2D from eight urban, publicly-funded, hospital-based clinics, we highlighted several findings in relation to the cardiometabolic risk profiles and quality of care. More than 90% of the cohort were either high- or very high-risk for cardiorenal diseases, showing no significant difference between the Diabetes specialist and General medicine clinics. The present cohort also had high rates of general and central obesity, especially in women with T2D. Of note, one in five patients attained ≥2 treatment targets (HbA_1c_<7% (53 mmol/mol), BP<130/80 mmHg, and LDL-C<1.8 mmol/L), showing a higher proportion among those either with prior ASCVD or managed in the General medicine clinics. Although statin coverage was >90%, there was suboptimal attainment of LDL-C targets even among patients with prior ASCVD, wherein fewer than 15% of them attained LDL-C<1.4 mmol/L. Despite having a high proportion of at-risk patients, use of newer guideline-directed medical therapy (GDMT) such as SGLT2 inhibitors and GLP1-RAs was suboptimal, especially among those managed in the General medicine clinics.

Compared with previous hospital-based studies that were conducted in 2008 and 2013, the control of glycaemia among patients with T2D in the present study has modestly improved [31,32]. About one-third of the present cohort attained HbA_1c_ <7% (53 mmol/mol), which was consistent with T2D populations in other middle-income countries such as China and India [33]. However, this was lower than what had been reported in high-income countries, which ranged between 45% and 80% [33]. The selection of HbA_1c_ <7% (53 mmol/mol) as a treatment target in the present study could be debated. This target was based on the 2022 American Diabetes Association Standard of Medical Care [27] and our local guideline [23]. In real-world practice, the HbA_1c_ target can be individualized between 7% (53 mmol/mol) and 8.5% (69 mmol/mol) according to the patient’s age, comorbidities, and hypoglycaemia risk [27,34]. On the other hand, therapeutic inertia could contribute to suboptimal control of glycaemia. In a retrospective analysis of the Malaysian primary care-based National Diabetes Registry, among non-insulin-treated patients with T2D and HbA_1c_ ≥8% (64 mmol/mol), 54% had delayed treatment intensification with a median time of 13 months [35].

Obesity, hypertension, and dyslipidaemia can interact with hyperglycaemia in the development and progression of cardiorenal diseases, cancer, and other microvascular complications [2]. In the present cohort, three-quarters had general obesity with a mean BMI of 30 kg/m^2^ and >90% of women had central obesity. These findings were consistent with a multinational CAPTURE study which involved a predominantly White population [36]. According to the 2019 population-based Malaysian National Health and Morbidity Survey, 50% of adults had a BMI of ≥25 kg/m^2^, being more common in women than in men [1]. Indeed, the mean BMI among patients with T2D has increased from 28 kg/m^2^ in DiabCare 2008 [31] to 29 kg/m^2^ in DiabCare 2013 [32], and 30 kg/m^2^ in the present cohort.

Achieving hypertension control has been challenging wherein only 23% of the present cohort reported having BP <130/80 mmHg. Although the presence of multiple comorbidities among patients at hospital-based clinics was common and could be associated with suboptimal control, the proportion of patients at primary care clinics attaining BP ≤135/75 mmHg, who tended to have fewer comorbidities, was similar at 26% [37]. One possibility was white-coat hypertension as clinic BP, but not home BP, was recorded. Other potential reasons include the lack of disease awareness, infrequent home BP monitoring, and suboptimal treatment adherence due to polypharmacy, medication side effects, and self-care behaviour [2,38]. In addition, salt intake is an important determinant of BP. Alarmingly, 79% of adults in Malaysia reported consuming >5 grams (1 teaspoon) daily, which was beyond the recommended intake by the World Health Organization [39].

We report that one in four high- and very high-risk patients had adequate control of LDL-C level (<1.8 mmol/L), which is consistent with other Asian countries/regions. In a large retrospective cohort of >100,000 high-risk patients with diabetes in Korea, the mean LDL-C level was 2.9 mmol/L and 12% attained LDL-C <1.8 mmol/L [40]. In a multinational SUrvey of Risk Factors (SURF) study, the proportion of high-risk patients attaining LDL-C <1.8 mmol/L was 15% in China and Taiwan, compared with 33% and 35% in European and Middle-Eastern countries [41]. Future analysis by the type and dose of statin therapy (high- versus moderate intensity), as well as the different combinations of lipid-lowering therapy, may provide insights on how to close these gaps in care. The lack of patient-provider communication on the safety of statin therapy such as muscle symptoms, may also affect patient treatment adherence [42]. In a meta-analysis of 19 randomized clinical trials, statin therapy was associated with an absolute excess rate of 6–16 events per 1000 person-years of muscle symptoms during year 1 [43]. There was no significant excess risk during subsequent years [43]. Indeed, 90% of all reports of muscle symptoms among statin-treated patients were not due to statin therapy [43]. Apart from the aforementioned patient-level factors, the lack of a reliable cardiovascular risk stratification tool and therapeutic inertia could be associated with suboptimal risk-based LDL-C management [44].

Our findings indicate that one-third of the present high-risk cohort were treated with SGLT2 inhibitors, with a much lower proportion of 13% in General medicine clinics. The latter is consistent with other reports with similar time periods from the US [45] and the CAPTURE study [36]. Use of GLP1-RAs was only 4% among patients with ASCVD, reflecting the lack of availability of this class of GDMT. The uptake of these GDMT may change with the recent updates to consensus guidance that now recommend an SGLT2 inhibitor or GLP1-RA as first or second-line treatment in patients with either high-risk T2D or cardiorenal diseases [23,24,34,46]. The influence of these consensus updates on real-world practice is of interest and hence, the present study provides a benchmark for quality improvement. On the other hand, access and availability of SGLT2 inhibitors and GLP1-RAs depend on the purchasing decisions, medication quota, and prescribing rights in individual publicly-funded hospitals. For instance, at the time of the TARGET-T2D study, SGLT2 inhibitors were not approved for use at General medicine clinics and hence, patients who were indicated for treatment would need to be referred to Endocrinologists.

The major strength of the present study is the shared protocol for standardized data collection and quality assurance at eight publicly-funded tertiary care hospitals. In addition, use of a structured care record form, established data definitions, and an electronic data capture system with quality control may be key measures for periodic performance tracking and identification of gaps in care [2]. Importantly, our hospital-based data will complement the findings from the National Diabetes Registry which involves publicly-funded primary care clinics. Taken together, we are hopeful that our data will offer an impetus for the improvement of care standards over time among patients with T2D in Malaysia.

We acknowledge several study limitations. First, due to the pragmatic nature of the present study, there was potential selection bias due to the enthusiasm of participating study sites. Patients who were younger, with shorter disease duration, and less complex diseases might be under-represented, biasing our findings to those with more severe disease. Second, given that all study sites were urban publicly-funded hospitals in the Greater Kuala Lumpur region, there is limited generalizability to patients with T2D living in rural areas, patients managed in private healthcare facilities, and on a nationwide level. Last, given that not all study sites had electronic health records systems and manual data extraction was necessary, we limited the number of data collected in the real-world practice by excluding questionnaires on hypoglycaemia risk and patient-reported outcomes.

In conclusion, compared with previous audits (although with different hospital-based patient groups), we have reported modest improvement in cardiometabolic risk factors and treatment target attainment in the public hospital setting. Given finite resources, our data highlight potential gaps in care which can facilitate effective resource allocation. To address the epidemic of T2D in Malaysia, the present TARGET-T2D study is well-positioned to enable future data collection on a nationwide level, monitoring of trends, and longitudinal evaluation of health outcomes.

## Supporting information

S1 File (DOCX)
